# Autofluorescence is a Reliable *in vitro* Marker of Cellular Senescence in Human Mesenchymal Stromal Cells

**DOI:** 10.1038/s41598-019-38546-2

**Published:** 2019-02-14

**Authors:** Alessandro Bertolo, Martin Baur, Julien Guerrero, Tobias Pötzel, Jivko Stoyanov

**Affiliations:** 1 Swiss Paraplegic Research, Nottwil, 6207 Switzerland; 2 Cantonal Hospital of Lucerne, Lucerne, 6000 Switzerland; 3 Swiss Paraplegic Centre, Nottwil, 6207 Switzerland; 4 Department of Biomedicine and Tissue Engineering, University of Basel Hospital, Basel, 4031 Switzerland; 5 Institute for Surgical Technology and Biomechanics, University of Bern, Bern, 3014 Switzerland

## Abstract

Mesenchymal stromal cells (MSC) are used in cell therapies, however cellular senescence increases heterogeneity of cell populations and leads to uncertainty in therapies’ outcomes. The determination of cellular senescence is time consuming and logistically intensive. Here, we propose the use of endogenous autofluorescence as real-time quantification of cellular senescence in human MSC, based on label-free flow cytometry analysis. We correlated cell autofluorescence to senescence using senescence-associated beta-galactosidase assay (SA-β-Gal) with chromogenic (X-GAL) and fluorescent (C_12_FDG) substrates, gene expression of senescence markers (such as p16^INK4A^, p18^INK4C^, CCND2 and CDCA7) and telomere length. Autofluorescence was further correlated to MSC differentiation assays (adipogenesis, chondrogenesis and osteogenesis), MSC stemness markers (CD90/CD106) and cytokine secretion (IL-6 and MCP-1). Increased cell autofluorescence significantly correlated with increased SA-β-Gal signal (both X-GAL and C_12_FDG substrates), cell volume and cell granularity, IL-6/MCP-1 secretion and with increased p16^INK4A^ and CCND2 gene expression. Increased cell autofluorescence was negatively associated with the expression of the CD90/CD106 markers, osteogenic and chondrogenic differentiation potentials and p18^INK4C^ and CDCA7 gene expression. Cell autofluorescence correlated neither with telomere length nor with adipogenic differentiation potential. We conclude that autofluorescence can be used as fast and non-invasive senescence assay for comparing MSC populations under controlled culture conditions.

## Introduction

Human mesenchymal stromal cells (MSC) are multipotent cells with the ability to replicate^1,2^ and differentiate into several mesodermal cell lineages, such as adipocytes, chondrocytes, myocytes and osteoblasts^3^. Furthermore, MSC have shown broad and extensive immunomodulatory effects^4,5^, which place MSC in a relevant position for cell-based therapies and tissue engineering approaches. Currently, MSC are involved in clinical trials as a therapy for immune-related diseases (such as graft versus host disease)^6,7^, bone and cartilage diseases, cardiovascular diseases and neurological diseases^8,9^. Although most of these studies are still phase I or II trials (according to ClinicalTrials.gov), promising results are already emerging. For instance, in the treatment of traumatic spinal cord injury, multiple administration of MSC improved motor function in patients not responding to standard therapy^10^. The ability of MSC to perform such tasks depends on the proteins they express and secrete. It has been shown that the secretome profile of MSC depends remarkably on the progression of cellular senescence^11^, potentially influencing and altering outcomes of the therapies.

Cellular senescence is a complex and possibly irreversible state occurring during cell and tissue ageing^12^. Senescence is accelerated by several factors – oxidative stress, DNA damage, telomere shortening and oncogene activation^13^ – and it is seen in part as an anti-tumorigenic process which halts dividing cells and, in association with apoptosis, prevents their potential malignant transformation^14^. Senescent cells express ligands and adhesion molecules that signal to natural killer and other immune cells to attack them^15^. This normally stimulates surrounding progenitor cells to regenerate the compromised tissue^13^. However, increased number of senescent cells is associated to decreased tissue regeneration capacity and life expectancy, and their elimination in a mouse model resulted in increased lifespan^16^. This identifies cellular senescence as an ideal target for the development of new anti-ageing therapies. Nevertheless, interventions and detection of senescent cells, both *in vitro* and *in vivo*, need further optimizations because of the complicated nature of the ageing process.

Senescence is a multifactorial process and the heterogeneity of cell populations can hinder the identification of specific markers. The most commonly used laboratory assay for the detection of cellular senescence is senescence-associated beta-galactosidase (SA-β-Gal)^17^, however the method has some limitations. Confluency, serum starvation and operator bias influence the assay outcomes with false positive results^18^. False negatives are also an issue – where some cells enter senescence without detectable SA-β-Gal activity^19^. Several other markers – such as oxidative status^20^ – can be used to identify cellular senescence, nonetheless their determination is time consuming and technically difficult to assess in living tissues. Therefore, development of readily available biomarker and the development of a sensitive and easy-to-perform assay to identify cellular senescence would provide major benefits in a rapidly evolving and challenging field such as regenerative medicine.

The first observations of autofluorescence were reported over 100 years ago^21^, as a spontaneously occurring phenomenon due to the accumulation within the cytoplasm of endogenous molecules with fluorophore-like properties. In senescent cells, under the pressure of oxidative stress, the level of autofluorescence increases because the aggregation within the cytoplasm of lipofuscin and lipofuscin-like compounds^22^. Analysis of cellular autofluorescence can be quickly and extensively used as it relies on common flow cytometry instruments (or fluorescent microscopy) which detect excitation of endogenous fluorophores produced by cell constituents, such as oxidative chain respiratory proteins (nicotinamide adenine dinucleotide (NADH), flavin adenine dinucleotide (FAD) and cytochrome C), structural proteins (collagen and elastin), vitamins (riboflavin and thiamine) and undegredable waste material (lipofuscin)^21^. Lipofuscin has been referred to as the “age pigment”^23^, as it accumulates within the cytoplasm with age and correlates with the rate of ageing^24^. Furthermore, riboflavin has already been recognized as a biomarker associated selectively with epithelial cancer stem cells^25^, while fluorescence has been proposed as a non-invasive indicator of embryonic stem cell differentiation state, to distinguishing pluripotent from more mature cells^26^.

Based on the above facts, we speculated that cellular autofluorescence would be an ideal candidate to test for the purpose of tracking senescence in MSC. The co-localization of lipofuscin and SA-β-Gal activity in senescent cells both *in vitro* and *in vivo* has been demonstrated in archival tissues, supporting the idea of using lipofuscin as biomarker for cellular senescence^27^, however no study has been conducted to elucidate whether the autofluorescence of MSC could be linked to measures of cellular senescence.

Cellular senescence has been successfully assessed not only by SA-β-Gal assay with chromogenic (X-GAL)^17^ and fluorescent (C_12_FDG)^28,29^ substrates, but also by cell size^30^ and granularity^31^, secretion of senescence-associated cytokines (IL-6 and MCP-1)^32^, gene expression of cell cycle regulators associated to cell senescence (p16^INK4A^, p18^INK4C^, p21^CIP1^, E2F1, ANKRD1, CCND2, CDC2 and CDCA7)^33–36^ and telomere length^37^. Variations in MSC stemness linked to cell senescence are monitored by surface markers (CD90 and CD106)^20,38^ and differentiation potential by adipogenic, chondrogenic and osteogenic assays^39^. In the present study, we tested the suitability of an autofluorescence profile of bone marrow-derived MSC measured by flow cytometry, as a tool for a rapid and non-invasive prediction of MSC senescence in correlation with the above mentioned markers for senescence, stemness and differentiation. We also included in the study three different culture conditions and extended our analysis to adipose-derived MSC and peripheral blood lymphocytes.

## Results

### Correlation of cellular senescence to autofluorescence in mesenchymal stromal cells (MSC)

**Figure 1:**
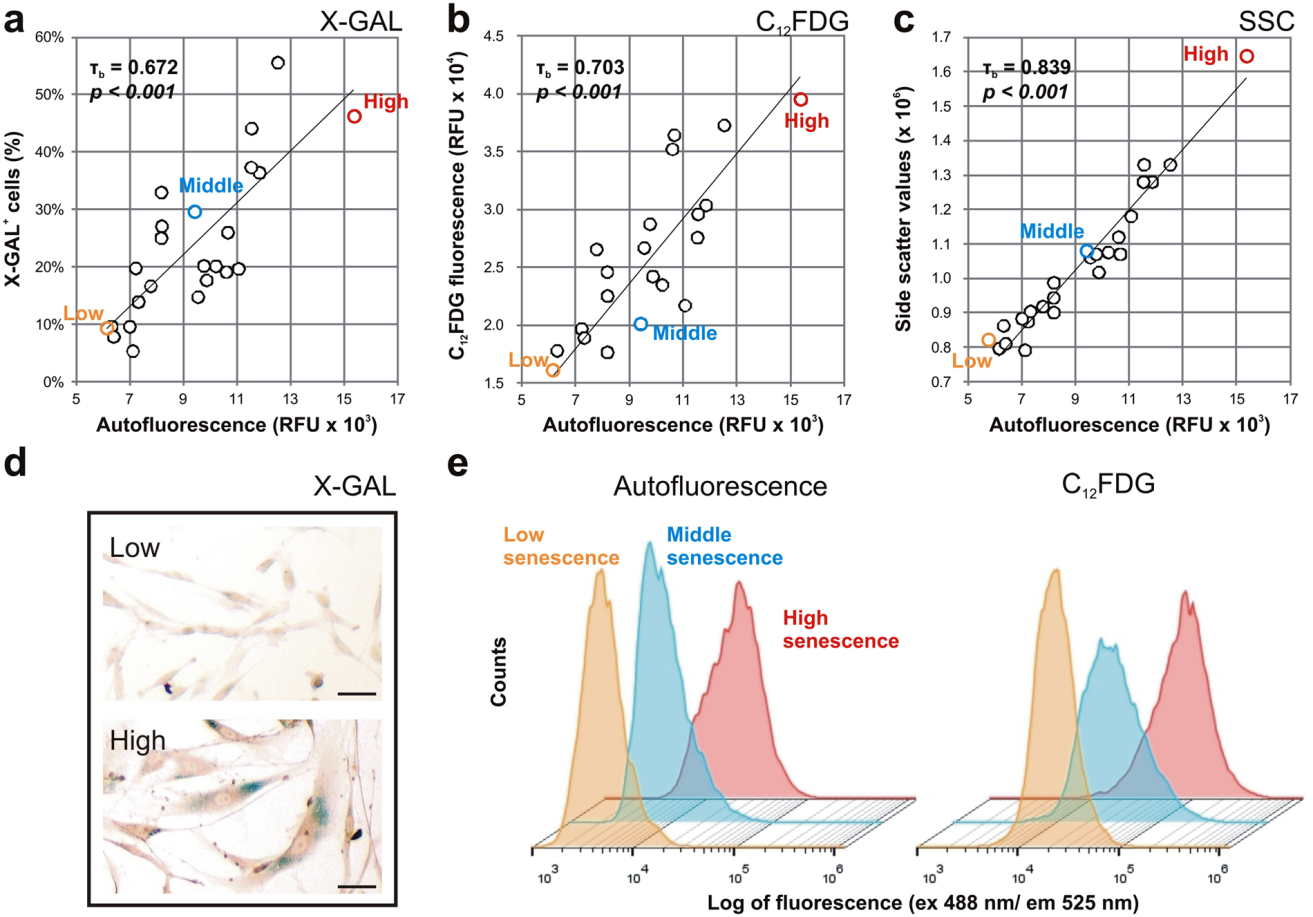
Correlation of MSC autofluorescence with senescence-associated markers. The autofluorescence of MSC (n = 24) correlated positively to SA-β-Gal assay, measured by (**a**) chromogenic- (X-GAL) and (**b**) fluorogenic– (C_12_FDG) substrates, and also correlated to (**c**) side scatter values (SSC). Autofluorescence, C_12_FDG and SSC values are represented as the geometric mean of the population, while X-GAL values as percent of positive cells in the MSC population. Representative (**d**) X-GAL stainings and (**e**) flow cytometry analysis, with and without C_12_FDG, are shown for samples of low, middle and high senescent populations. Scale bar = 50 µm.

In order to characterize cellular senescence, bone marrow isolated MSC were initially categorized by their senescence-associated beta-galactosidase (SA-β-Gal) activity, evaluated with chromogenic (X-GAL, Fig. 1a) and fluorescent substrates (C_12_FDG, Fig. 1b). The proportion of X-GAL positive cells, as a percent of the total population, significantly increased with cellular autofluorescence (τ_b_ = 0.672, *p* < *0.001*), as well as the fluorescence intensity of MSC population stained with C_12_FDG (τ_b_ = 0.703, *p* < *0.001*). Interestingly, we also observed a high degree of correlation between cellular granularity (Fig. 1c) – represented by side scatter values – and cellular autofluorescence of MSC (τ_b_ = 0.839, *p* < *0.001*). Representative images of samples characterized by low, middle and high degree of senescence stained by X-GAL (Fig. 1d) and C_12_FDG (Fig. 1e) are shown.

**Figure 2:**
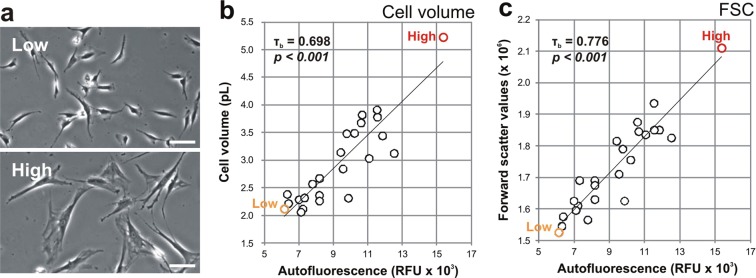
Correlation of MSC autofluorescence with cell size. Morphological differences of low and high senescent MSC cultures are shown. (**a**) Scale bar is 50 µm. The autofluorescence of MSC (n = 24) was positively correlating with (**b**) cell volume and (**c**) forward scatter values (FSC). Autofluorescence and FSC values are represented as the geometric mean of the population, while cell volume as the mean.

Senescent MSC show well defined morphological alterations, such as enlarged and flattened aspect^40^ (Fig. 2a), and for this reason cellular autofluorescence was also correlated to their size, as a measure of cell volume (Fig. 2b) and forward scatter values (Fig. 2c). A strong and significant correlation was found between autofluorescence and both cell volume (τ_b_ = 0.698, *p* < *0.001*) and forward scatter values (τ_b_ = 0.776, *p* < *0.001*).

**Figure 3:**
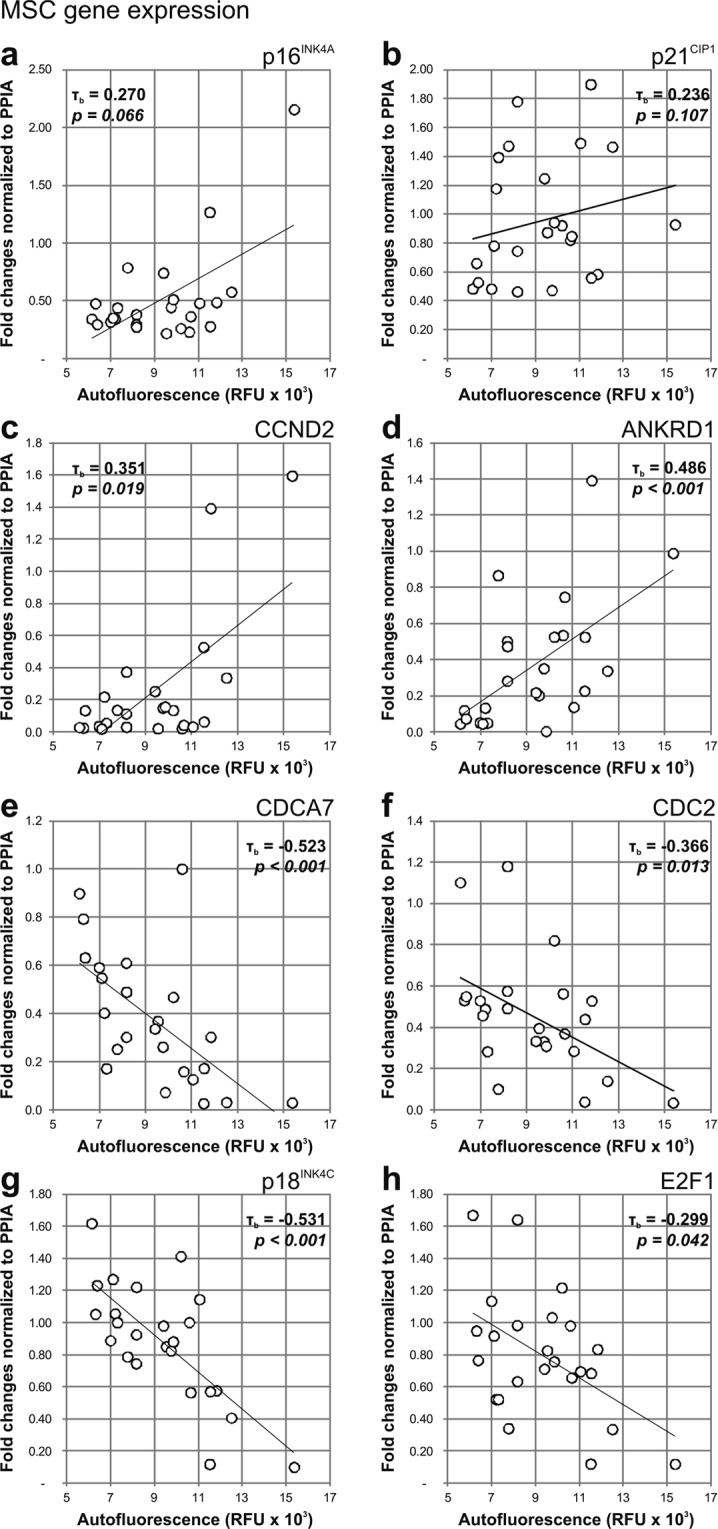
Correlation of MSC autofluorescence with the gene expression of senescence markers. The autofluorescence of MSC (n = 24) was positively correlating to the gene expression levels of (**a**) p16^INK4A^, (**b**) CCND2, (**c**) ANKRD1, but not of (**d**) p21^CIP1^. Conversely, autofluorescence of MSC was negatively correlating to (**e**) CDCA7, (**f**) CDC2, (**g**) p18^INK4C^ and (**h**) E2F1. Gene expression was normalized to PPIA, and each sample represents the mean expression. Autofluorescence values are represented as the geometric mean of the population.

To further explore the dynamics of MSC during the process of cellular senescence, we evaluated other senescence-associated markers such as changes in gene expression, cytokine secretion and telomere length. At the gene expression level, we observed a low and non-significant correlation between the expression of p16^INK4A^ (Fig. 3a; τ_b_ = 0.270, *p* = *0.066*) and p21^CIP1^ (Fig. 3b; τ_b_ = 0.236, *p* = *0.107*) by MSC and cellular autofluorescence. The expression of CCND2 (Fig. 3c; τ_b_ = 0.351, *p* = *0.019*) and ANKRD1 (Fig. 3d; τ_b_ = 0.486, *p* < *0.001*) was also increasing with cell autofluorescence, but significantly. On the other hand, the expression of CDCA7 (Fig. 3e; τ_b_ = −0.523, *p* < *0.001*), CDC2 (Fig. 3f; τ_b_ = −0.366, *p* = *0.013*), p18^INK4C^ (Fig. 3g; τ_b_ = −0.531, *p* < *0.001*) and E2F1 (Fig. 3h; τ_b_ = −0.299, *p* = *0.042*) was significantly and negatively correlated to cellular autofluorescence.

**Figure 4:**
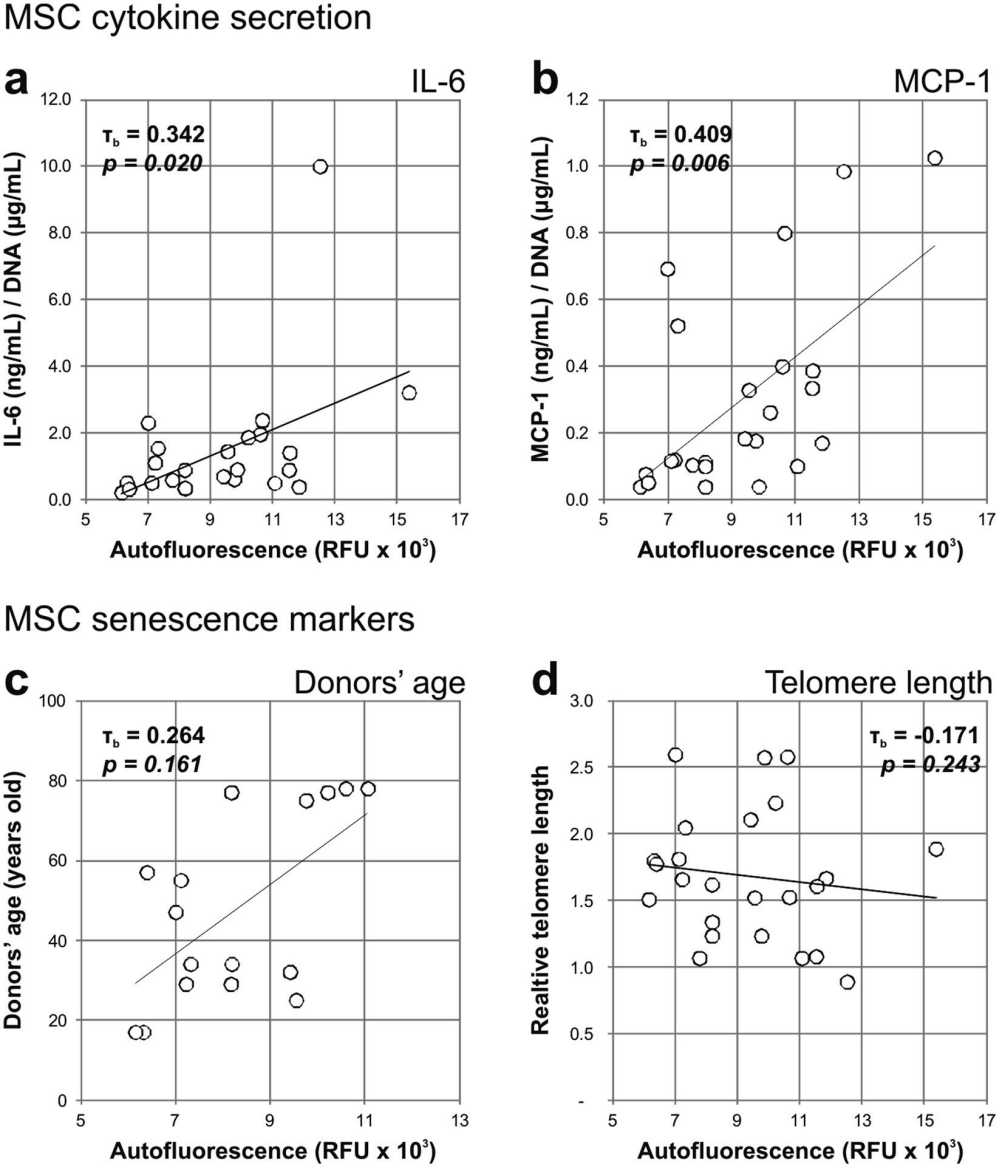
Correlation of MSC autofluorescence with cellular senescence markers. The autofluorescence of MSC (n = 24) was positively correlating to the mean cell secretion in culture media of (**a**) IL-and (**b**) MCP-1. No correlation between cell autofluorescence and (**c**) donors’ age (only early passage MSC, from P1 to P4, n = 16), and (**d**) telomere length was observed. Autofluorescence values are represented as the geometric mean of the population.

Changes in the secretion of IL-6 (Fig. 4a) and MCP-1 (Fig. 4b) by MSC were determined by ELISA analysis of the culture media, after three days incubation with cells. Increase in the secretion of IL-6 was significantly and lowly correlated to increased MSC autofluorescence (τ_b_ = 0.342, *p* = *0.020*), as well as MCP-1 (τ_b_ = 0.409, *p* = *0.006*).

Alternative variables which we considered that might be involved in replicative cellular senescence were increasing donors’ age (Fig. 4c) and telomere shortening (Fig. 4d). Including only low passage MSC populations (from P1 to P4), we observed a low degree and non-significant correlation between donors’ age and cellular autofluorescence (τ_b_ = 0.264, *p* = *0.161*). Furthermore, no correlation between cell autofluorescence and the relative length of telomeres was found (τ_b_ = 0.171, *p* = *0.243*).

### Correlation of MSC differentiation potentials and autofluorescence

**Figure 5:**
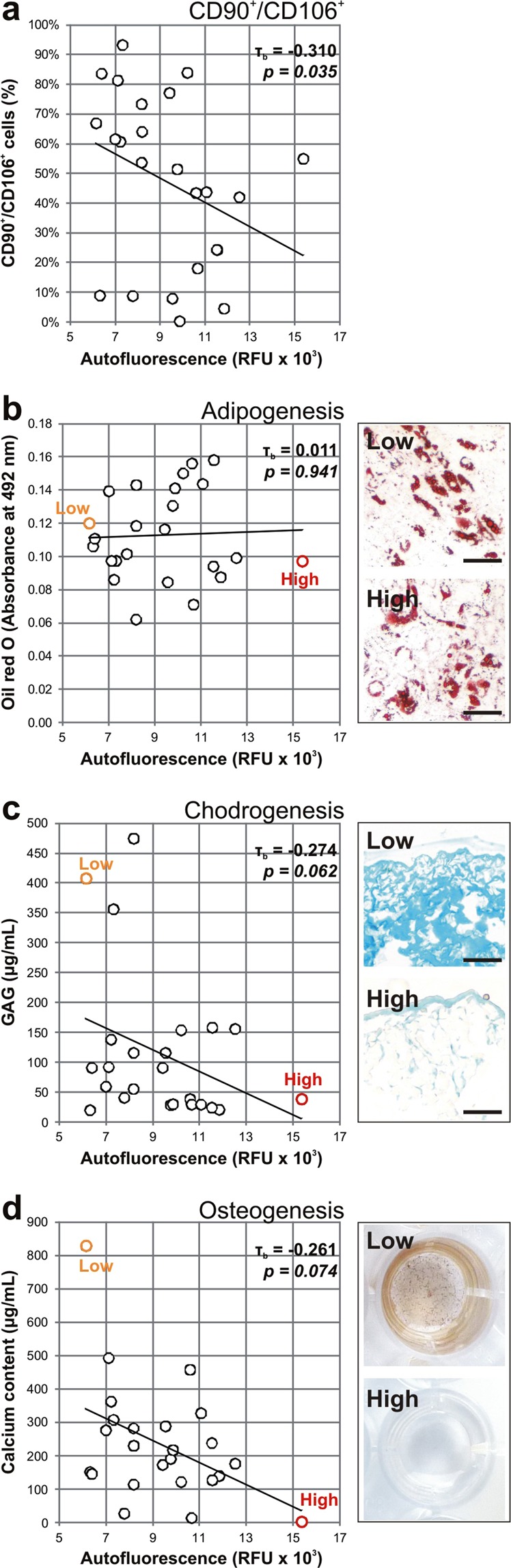
Correlation of MSC autofluorescence and cell differentiation potentials. The autofluorescence of MSC (n = 24) was weakly correlating to the proportion of CD90+/CD106+ cells within the population. (**a**) The adipogenic potential of MSC had no correlation with cell autofluorescence (**b**), while chondrogenic (**c**) and osteogenic (**d**) potentials were negatively correlated to cell autofluorescence. Autofluorescence values are represented as the geometric mean of the population. Images of representative low and high senescent populations are shown beside the graphs. Differentiated cultures were stained for fat vacuole formation in red (Oil red O), accumulation of proteoglycan in blue (alcian blue staining) and calcium in black (von Kossa staining) respectively. Scale bar = 100 µm.

During *in vitro* senescence, MSC markers have been described to decrease^20,38^. Here we characterized MSC by flow cytometry analysis based on the positive expression of mesenchymal stromal cell markers CD90 and CD106 (Fig. 5a), and observed a low degree and significant correlation between cellular autofluorescence and the proportion of double positive cells in MSC populations (τ_b_ = −0.310, *p* = *0.035*).

We also correlated cellular autofluorescence to the ability of MSC to differentiate towards adipogenic (Fig. 5b), chondrogenic (Fig. 5c) and osteogenic (Fig. 5d) lineages. There was no correlation between the values for adipogenic differentiation and MSC autofluorescence (τ_b_ = 0.011, *p* = *0.941*), while increased cellular autofluorescence was negatively correlating to chondrogenic (τ_b_ = −0.274, *p* = *0.062*) and osteogenic (τ_b_ = −0.261, *p* = *0.074*) potentials of MSC. Images of representative low and high senescent populations are shown beside the graphs. Adipogenic cultures were stained for fat vacuole formation (in red; Oil red O staining), chondrogenic cultures for accumulation of proteoglycan (in blue, alcian blue staining) and osteogenic cultures for calcium deposition (in black, von Kossa staining).

### Correlation of cellular senescence and autofluorescence over increasing passages in MSC cultures

**Figure 6:**
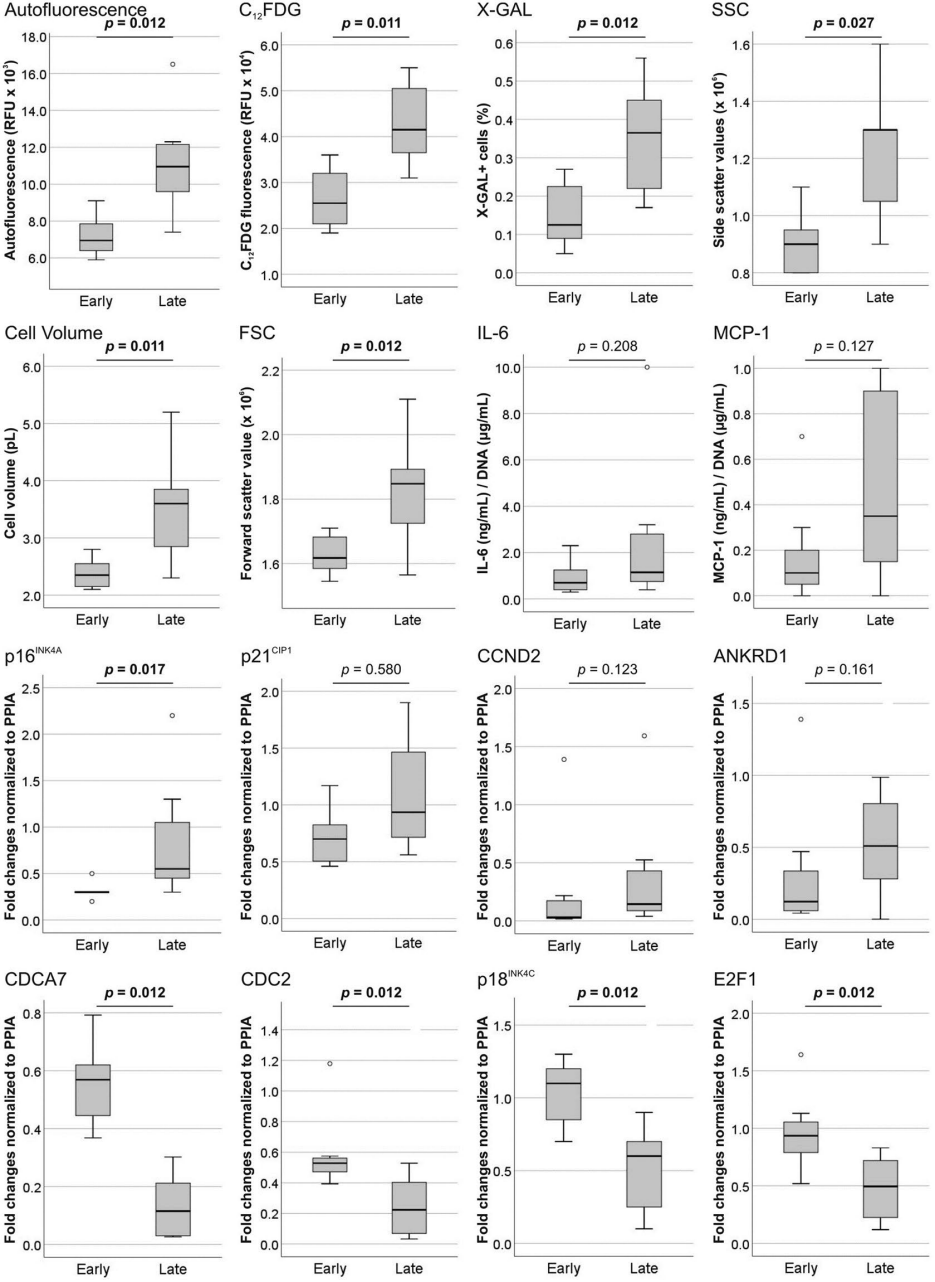
Changes in MSC senescence markers occurring with prolonged *in vitro* culture. The expression of senescence markers was compared in MSC (n = 8) at early (P1 to P4) and late (P6 to P15) passages. The analysis was conducted by comparing cellular autofluorescence, fluorescent (C_12_FDG) and chromogenic (X-GAL) SA-β-Gal activity, cellular granularity (SSC), cell size - as a measure of cell volume and forward scatter values (FSC) -, telomere length, the proportion CD90/CD106 positive cells (stemness markers), gene expression (CDCA7, CDC2, p18^INK4C^, E2F1, p16^INK4A^) and the differentiation potential of MSC (adipogenic, chondrogenic and osteogenic potentials). Non-parametric test (Mann-Whitney U-test) was used to determine the significance levels.

Changes in cellular senescence were assessed by comparing early (P1 to P4) and late (P6 to P15) passages of MSC (Fig. 6). Compared to early passages, late passages had significantly increased cellular autofluorescence (*p* = 0.012), fluorescent (C_12_FDG, *p* = 0.011) and chromogenic (X-GAL, *p* = 0.012) SA-β-Gal activity, and cellular granularity (SSC, *p* = 0.027). Cell size, as a measure of cell volume (*p* = 0.011) and forward scatter values (*p* = 0.012), were also significantly increasing with passages. While the proportion of cells positive to CD90/CD106 staining decreased significantly (*p* = 0.028), telomere length was decreasing non-significantly with time in culture. MSC gene expression of CDCA7, CDC2, p18^INK4C^ and E2F1 (for all four, *p* = 0.012) was significantly decreasing at late passages compared to early ones. On the other hand, the expression of p16^INK4A^, CCND2, ANKRD1 and p21^CIP1^ was increasing with cell passaging (data not shown), however only changes in p16^INK4A^ expression were significant (*p* = 0.017). The adipogenic potential of early and late passage MSC was equivalent, while chondrogenic (*p* = 0.035) and osteogenic (*p* = 0.012) potentials were significantly reduced at late passages. Although not significantly, the secretion of IL-6 and MCP-1 was increasing at later passages (data not shown).

**Figure 7:**
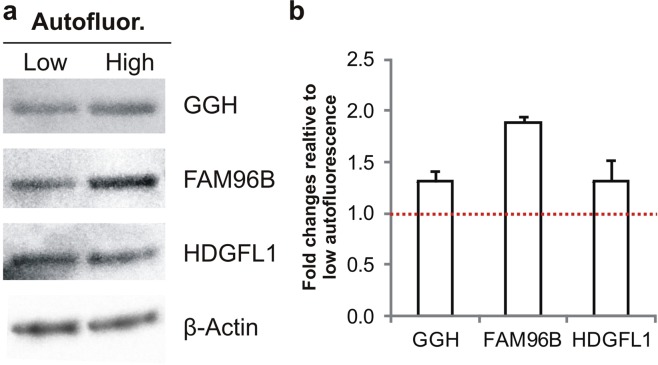
Western blot analysis of lipofuscin-related proteins. The protein expression of GGH, FAM96B and HDGFL1 was compared in low (early passages) and high (late passages) autofluorescent MSC. (**a**) Data was quantified and normalized to β-actin expression. (**b**) Results are represented as fold changes compared to the respective low autofluorescence MSC (red dotted line). (n = 3; values represent the mean ± SEM).

Protein extracts from early (low autofluorescence) and late (high autofluorescence) passages MSC were used to assess changes in lipofuscin expression with GGH, FAM96B and HDGFL1 antibodies^41^ by western blot (Fig. 7a). Quantification of bands (Fig. 7b) showed that - in comparison to low autofluorescent MSC - high autofluorescent MSC (n = 3) had higher expression of GGH (1.3-fold), of FAM96B (1.9-fold) and of HDGFL1 (1.3-fold).

### Correlation of SA-β-Gal activity and autofluorescence in different culture conditions

**Figure 8:**
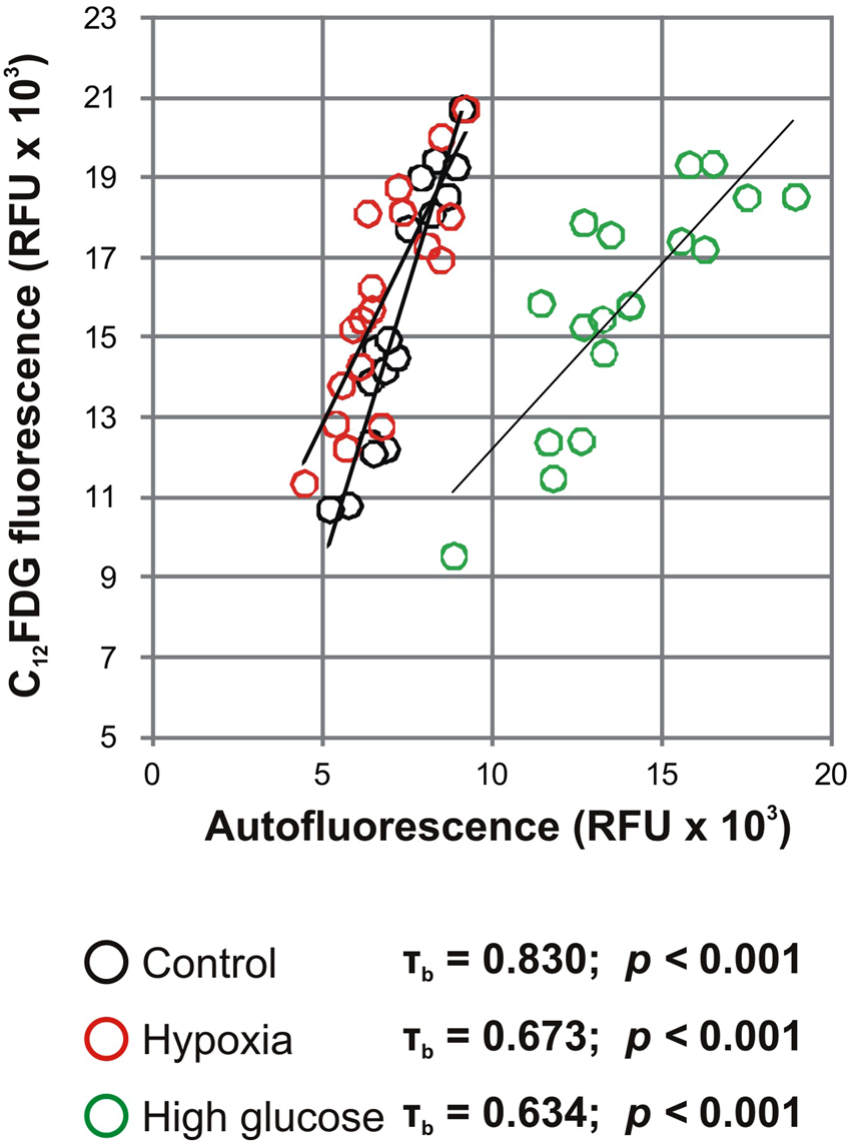
Correlation of MSC autofluorescence with SA-β-Gal activity under different culture conditions. MSC were grown either in high glucose DMEM/Ham’s F12 (4.5 g/L glucose) and normoxia (~21% O_2_), or in growing medium DMEM/Ham’s F12 (3.1 g/L glucose) and hypoxia (5% O_2_). For each donor (n = 6), cells in the respective cultures were sampled after 3, 5 and 7 days and results were compared to growing medium in normoxia (control).

We evaluated the influence of glucose concentration and oxygen levels on the correlation between cellular autofluorescence and SA-β-Gal. MSC (n = 6) were grown and sampled after 3, 5 and 7 days in high glucose DMEM/Ham’s F12 (4.5 g/L glucose, Amimed) and normoxia (~21% O_2_), growing medium DMEM/Ham’s F12 (3.1 g/L glucose) and hypoxia (5% O_2_), and growing medium DMEM/Ham’s F12 (3.1 g/L glucose) and normoxia (control). In the control culture the proportion of C_12_FDG positive cells strongly correlated with cellular autofluorescence (τ_b_ = 0.830, *p* < *0.001*), and to a lower extent under hypoxic (τ_b_ = 0.673, *p* < *0.001*) and high glucose(τ_b_ = 0.634, *p* < *0.001*) conditions (Fig. 8). Interestingly, while SA-β-Gal intensity was similar in all groups, MSC autofluorescence of cells exposed to high glucose levels was twofold higher compared to control (*p* < *0.001*), but unaltered in hypoxia.

### Correlation of SA-β-Gal activity and autofluorescence in other cell types

**Figure 9:**
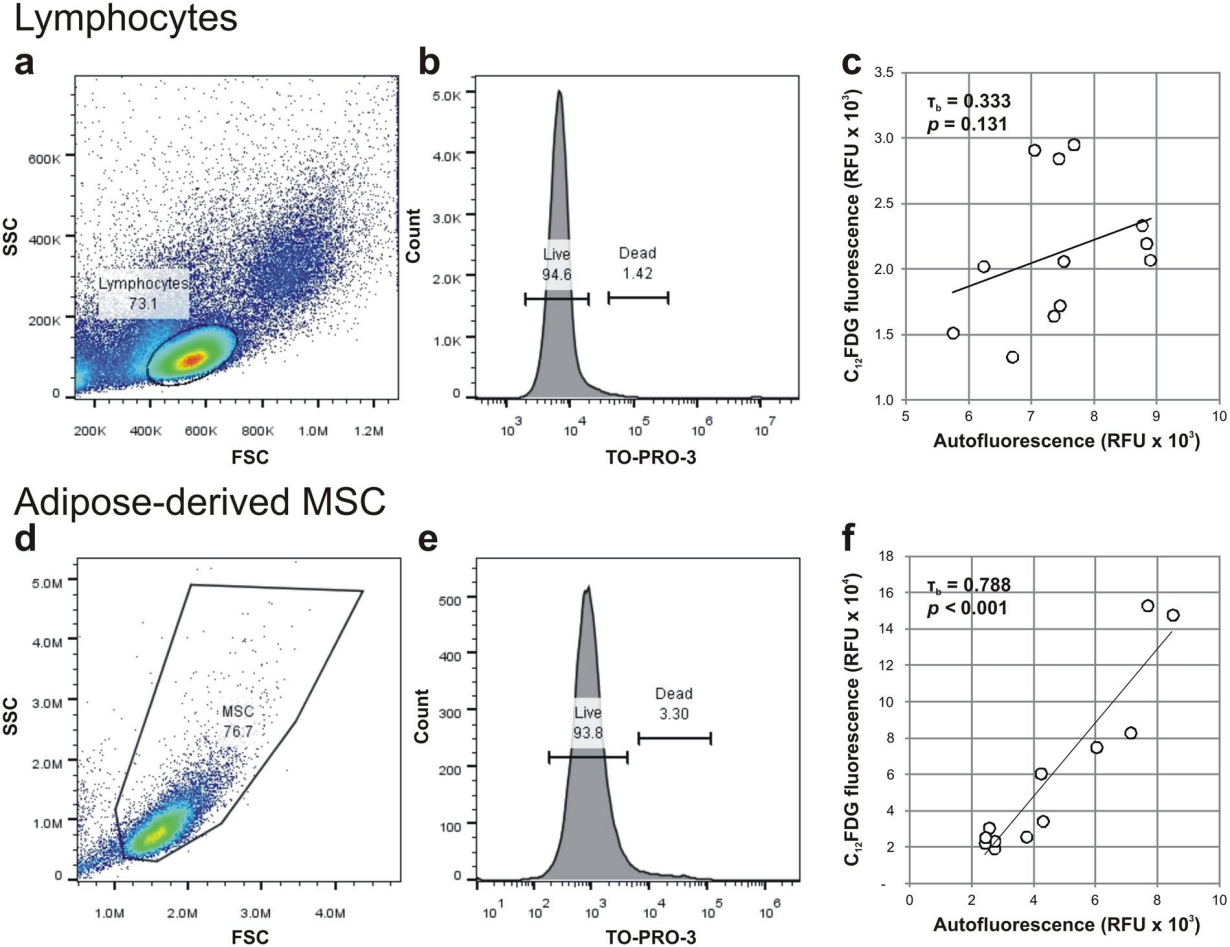
Correlation of cellular autofluorescence and SA-β-Gal activity in other cell types. The correlation between autofluorescence and SA-β-Gal activity (with fluorescent substrate C_12_FDG) was tested in (**a–c**) lymphocytes (n = 12) and (**d–f**) adipose-derived MSC (ADSC; n = 12). Gated lymphocytes (**a**) and ADSC (**e**) were selected base on their viability (**b**,**e**), and the correlation between cellular autofluorescence and (**c**,**f**) SA-β-Gal activity was established. Autofluorescence and C_12_FDG values are represented as the geometric mean of the population.

To evaluate if the correlation between SA-β-Gal activity and autofluorescence was cell-type specific, we performed the same analysis in two other cell types, namely peripheral blood lymphocytes (Fig. 9a) and adipose-derived MSC (Fig. 9d). In lymphocytes, after the exclusion of dead cells (Fig. 9b), C_12_FDG fluorescence intensity had low and non-significant correlation to autofluorescence (Fig. 9c; τ_b_ = 0.333, *p* = 0.131). On the contrary, in living adipose-derived MSC (Fig. 9e), C_12_FDG fluorescence intensity was strongly and significantly correlating to autofluorescence (Fig. 9f; τ_b_ = 0.870, *p* < *0.001*).

## Discussion

Ageing is an irreversible process in multicellular organism and only recently it has been mathematically proven to be unavoidable, by demonstrating the fine balance regulating accumulation of senescent cells in tissues and prevention of cancer cells proliferation^14^. However, it is not trivial to find the link between chronological age and biological senescence, and for individual organisms it requires empirical measurements. In this study, we showed the successful use of endogenous autofluorescence as a non-destructive tool to determine the *in vitro* age of mesenchymal stromal cells (MSC).

When cells become senescent *in vitro* they significantly change their phenotype. These changes also affect MSC deeply at a functional level. For instance, late passage MSC were clinically shown to be less effective in ameliorating graft-versus-host disease than early passage cells^7,42^. Therefore, a need exists to develop reliable and efficient tools to monitor the progression of senescence in tissues and cells – especially in the context of tissue regeneration – for the proper assessment of efficacy, patient inclusion criteria and regulatory/insurance issues.

Based on the literature, we speculated the involvement of lipofuscin in the autofluorescence observed by us, as it is mostly undegredable and accumulates only in senescent or slowly dividing cells, while proliferating cells dilute lipofuscin content with progressive divisions^43^. Indeed, Western blot analysis showed a significant higher accumulation of lipofuscin-related proteins - FAM96B and GGH - in highly autofluorescent MSC, compared to low ones. The different degree of antibody affinity to lipofuscin – which is a well-known molecule, but still elusive in its structure - might be the cause of the non-significant results with HDGFL1 antibody. Lipofuscin autofluorescence in MSC is localized within lysosomes^44^ and the increase in lysosomal mass occurring with senescence^45^, is partially reflected by increased expression of senescence-associated beta-galactosidase (SA-β-Gal)^17^. Considering that our results show increased autofluorescence and granularity correlating to MSC senescence, we assumed a direct link between autofluorescence and elevated lysosome content or activity, as a surrogate marker for SA-β-Gal activity^18^, which in turn can be used to assess, with a label-free method, cellular senescence in MSC.

Considering the strong reported links between lipofuscin autofluorescence and individual cell dysfunction, we presumed that ‘the missing link’ that will mark an advance in the field of high throughput detection of MSC senescence is the use of a sensitive, precise and easy to automate tool, such as flow cytometry. To validate such an approach, we demonstrated the strong association between autofluorescence and commonly used markers of senescent cells *in vitro*. Most importantly, we observed that increased expression of SA-β-Gal^17^ – measured both with chromogenic and fluorescent substrates – was strongly correlating with increased autofluorescence. Similarly, we also found a strong positive correlation with cell size of senescent MSC (as they turn into flat and hypertrophic cells), the expression of genes commonly expressed by senescent cells^33,34,36^ (p16^INK4A^, CCND2 and ANKRD1) and the increased secretion of factors involved in the senescence-associated secretory phenotype (SASP)^32^, MCP-1 and IL-6. On the other hand, expected negative correlations were observed between autofluorescence and MSC gene expression of E2F1, which controls cell cycle progression from G1 to S phase^35^, CDCA7, CDC2 and p18^INK4C^. Osteogenic and chondrogenic differentiation potentials of MSC were negatively correlated to autofluorescence, confirming previous findings of declining differentiation potentials after prolonged time in culture^30^. The expression of two stemness surface markers^20,38^ – CD106 (VCAM-1) and CD90 – here shown to be downregulated with cell senescence – showed only a weak correlation with autofluorescence.

Although telomere erosion is a well-established marker of cell senescence, inter-donor variability renders it an unreliable measure of MSC senescence^20,30,46^. Rather, “cell culture stress” such as oxidative stress (hyperphysiological levels of O_2_), variability between serum and media batches, and suboptimal culture factors has a more remarkable effect on cell division arrest^47^, where senescence results from. With the progression of senescence, MSC accumulate cell debris and excess of actin fibers promoting the formation of a granular cytoplasm^31^ – demonstrated by significantly higher scatter values – and thus increased autofluorescence. The same effect of cell debris accumulation and increased autofluorescence were evident not only in bone marrow derived MSC but also in adipose-derived MSC (ADSC). The absence of correlation between lymphocyte autofluorescence and SA-β-Gal activity only shows that such phenomena are cell type specific. Possibly, lymphocytes are too short-lived to accumulate the intracellular material which characterized cultured MSC and ADSC, or simply they have lower overall cytoplasm volume and a different ageing cell metabolism.

Despite the obvious advantages, there are some limitations in the use of autofluorescence. For example, the low correlation between some senescent markers - such as gene expression of p16^INK4A^ and p21^CIP1^ - and autofluorescence might be result of high inter-donor variability. In this study we used the most common laboratory practices to culture MSC, but we do not know if factors such as confluence and serum starvation, reported as limiting conditions for the SA-β-Gal assay, are likely to affect the autofluorescence signal. For instance, we observed that cell death affected autofluorescence outcome of MSC populations. Dead/dying cells had a significant lower autofluorescent levels compared to living cells and for this reason we included only living cells in our cell fluorimeter analysis. Also, similar autofluorescence changes observed with cellular ageing may be compromised from other perturbations, such as different media components. We observed that neither higher level of glucose in culture (4.5 g/L) nor hypoxia (5% O_2_) influenced the expression of SA-β-Gal (C_12_FDG fluorescence intensity), but rather affected cellular autofluorescence. However, the correlation between cellular autofluorescence and C_12_FDG fluorescence intensity was consistent among samples grown in the same culture conditions. We concluded that autofluorescence, as an indicator of MSC senescent level, measured in different culture settings cannot be compared directly. Furthermore, given the variety of assays we used in this project to analyze autofluorescence, we had to make a choice and omitted other existing senescent markers, such as DNA damage response (DDR)^48^ – measuring the genomic damage occurring at non-telomeric sites – and cell autophagy^49^.

In conclusion, we demonstrated that autofluorescence is a prospective indicator of MSC senescent level in a controlled culture setting. Considering the declining functioning of MSC with age and prolonged *in vitro* culture, the request for reliable, fast and label-free cell characterization is fundamental for the assessment of impairment of tissue homeostasis and tissue repair, and ultimately the difference between successful regeneration or organ failures^50^. Despite the inter-donor heterogeneity of MSC populations, autofluorescence can be successfully used to indicate the overall potential of cell populations. The assessment of MSC quality in clinical applications requires documentation at every step between MSC extraction, expansion and administration. We believe that this label-free detection of cell senescence can contribute not only to a better understanding of the properties of MSC, but has the potential to become a standard MSC senescence marker. Building on our previous work on assessing MSC ageing and differentiation potential^20,30,51^, we conclude that the measurement of autofluorescence is an economic assay which can be used alone, or complementary to other tests, in order to improve research and the outcomes of cell-based therapies. In perspective, further development of autofluorescence as a validated senescence marker would allow improved sensitivity and specificity measurements in cells - as done with validated diagnostic biomarkers – but in order to so there is a need for standardized cell senescence reference biomarkers.

## Materials and Methods

All methods were performed in accordance with the relevant guidelines and regulations of this journal.

### Cell isolation and expansion

#### Bone marrow-derived MSC

Bone marrow (BM) samples were harvested from the iliac crest of donors undergoing spine surgery. The study was ethically approved by the ethics committee of canton Lucerne (study number: 730). Written informed consent was obtained from all participants and this procedure was also accepted by the approving body. MSC were isolated from BM of 16 donors (7 females and 9 males; average age: 48 years; range: 17–78 years). The BM aspirates were immediately resuspended in 3.8% sodium citrate and phosphate buffered saline (PBS, AppliChem - Axonlab, Baden, Switzerland) and then filtered through a 100 µm cell strainer to remove clots (Falcon - Faust, Schaffhausen, Switzerland). Mononuclear cells were separated by H-Lympholyte Cell Separation Media gradient centrifugation (density 1.077 g/mL; Cedarlane - Bio Concept, Allschwil, Switzerland) in Leucosep tubes (Huberlab, Reinach, Switzerland) at 800 *g* for 15 minutes, washed with PBS, centrifuged again at 210 *g* for 10 minutes and plated at a density of 1 × 10^5^ cells/cm^2^ in tissue culture flasks (TPP – Faust) in α-MEM (Amimed – Bio Concept), supplemented with 10% fetal bovine serum (FBS, Gibco – LuBioScience, Lucerne, Switzerland), (100 units/mL) penicillin/(100 mg/mL) streptomycin (Pen-Strep, Gibco), 2.5 µg/ml amphotericin B (Sigma, Buchs, Switzerland) at 37 °C in a humid atmosphere containing 5% CO_2_. After two days, non-adherent cells were discarded, whereas adherent cells were cultured in growing medium consisting of DMEM/Ham’s F12 (3.1 g/L glucose, Amimed), supplemented with 10% FBS, 1X Pen-Strep, 2.5 µg/ml amphotericin B and 5 ng/ml recombinant basic fibroblast growth factor (bFGF, Peprotech – LuBioScience), followed by media change three times per week. At 80% confluency, MSC were frozen and stored at −150 °C.

Frozen MSC (n = 24) at different passage number (ranging from P1 to P15; for 8 donors, both early and late passages were included) were thaw and cultured for two days (SA-β-Gal assay), three days (cytokine measurements) and four days (phenotypic characterization, RNA and DNA isolation) in growing medium. Cell volume was determined by Scepter cell counter (Millipore, Thermo Fisher Scientific, Zug, Switzerland).

In addition to the previously described growing medium, MSC isolated from six donors were also cultured in diverse conditions to compare their influence on cell autofluorescence. MSC were grown either in high glucose DMEM/Ham’s F12 (4.5 g/L glucose, Amimed) and normoxia (~21% O_2_), or in growing medium DMEM/Ham’s F12 (3.1 g/L glucose) and hypoxia (5% O_2_). For each donor (n = 6), cells in the respective cultures were sampled after 3, 5 and 7 days and results were compared to growing medium in normoxia (control).

#### Adipose marrow-derived MSC (ADSC)

Adipose tissue, in the form of liposuction or excision samples, was obtained from 6 healthy donors following informed consent and according to a protocol approved by the local ethical committee (EKBB, Ref. 78/07). All donors were females between 28 and 75 years of age. Finely minced excision samples or liposuction samples were digested with 0.075% collagenase type II (355 U/mg, Worthington, Lakewood, NJ, USA) for 60–90 min at 37 °C, as previously described^52^. After centrifugation at 190 *g* for 10 min, the lipid-rich layer was discarded and the cellular pellet was washed once with PBS. Red blood cells were lysed by incubation for 2 min in a solution of 0.15 M ammonium chloride, 1 mM potassium hydrogen carbonate (both Merck, Darmstadt, Germany) and 0.1 mM EDTA (Fluka Analytical, Sigma). The resulting SVF cells were then resuspended in α-MEM supplemented with 10% FBS, 1% HEPES, 1% Sodium pyruvate and 1X of Penicillin-Streptomycin-Glutamine solution (all three from Gibco) at 37 °C in a humid atmosphere containing 5% CO_2_. SVF cells were seeded at a density of 2 × 10^3^ cells/cm^2^ and cultured in medium supplemented with 5 ng/mL bFGF, serially replated in new dishes when reaching subconfluence, and then frozen at −150 °C.

Frozen ADSC (n = 12) at different passage number (for each donor, both early (P2-P3) and late passages (P16-P19) were included) were thaw and cultured for two days before performing the senescence-associated beta-galactosidase (SA-β-Gal) assay.

#### Lymphocytes

Venous blood was collected in S-Monovette containing sodium citrate for white blood cell isolation (Sarstedt, Sevelen, Switzerland). Donors (n = 12) were between 29 and 80 years of age (1/3 male and 2/3 female). Peripheral blood mononuclear cells (PBMC) were isolated by H-Lympholyte Cell Separation Media in a Leucosep tube by gradient centrifugation at 800 *g* for 20 min. The PBMC containing buffy coat was carefully retrieved and washed with PBS, followed by a centrifugation at 210 *g* for 10 min. The obtained pellet was resuspended in freezing media - 10% dimethyl sulfoxide (AppliChem), 45% RPMI-1640 medium (Amimed) and 45% FBS - and then frozen in a CoolCell (Biocision, Sigma) container. Ultimately, frozen cell suspension was transferred to a long term cryogenic storage (−150 °C Ultra-low Temperature freezer MDF-C2156VAN, Panasonic).

PBMC were thawed in a water bath at 37 °C and immediately diluted 8-fold with PBS +10% FBS. The suspension was centrifuged at 210 *g* for 10 min and cells (4 × 10^6^ cells/mL) were left to recover overnight in RPMI-1640 medium and 10% FBS, 1X Pen-Strep ad 2.5 µg/ml amphotericin B at 37 °C in a humid atmosphere containing 5% CO_2_. The next day, PBMC (specifically lymphocytes) were tested for their SA-β-Gal activity by flow cytometry.

### Phenotypic characterization of MSC

Following cell culture expansion, CD90 and CD106 positive MSC markers were analyzed by flow cytometry. Briefly, cells were incubated with CD90/Thy1-FITC (5E10; NBP1-96125, Novus Biological) and CD106-APC (Cat. No. 305809, BioLegend - LucernaChem, Lucerne, Switzerland) antibodies in PBS for 30 minutes at room temperature, washed and resuspended in PBS. Cell fluorescence was evaluated with CytoFLEX flow cytometer (Beckman Coulter Life Sciences, Nyon, Switzerland) and data were analyzed using FlowJo v.10.0 software (Treestar, Ashland, OR, USA).

### Senescence-associated beta-galactosidase (SA-β-Gal) assay

SA-β-gal activity of MSC was determined using chromogenic-^17^ and fluorescent-based^28,29^ methods, as described previously. In the chromogenic method, cells were fixed with 2% formaldehyde and 0.2% glutaraldehyde in PBS (both AppliChem) followed by incubation over night at 37 °C in a freshly prepared staining solution at pH 6.0 consisting of: 5 mM K_3_Fe(CN)_6_, 5 mM K_4_Fe(CN)_6_, 2 mM MgCl_2_, 150 mM NaCl, 30 mM citric acid/phosphate buffer (all AppliChem) and 1 mg/mL 5-bromo-4chloro-3-indolyls β-D-galactopyranoside (X-Gal, Sigma). Following PBS washing, cells were counterstained with haematoxylin (Molecular probes) and SA-β-Gal positive cells were manually enumerated with brightfield microscopy (Olympus, Volketswil, Switzerland) at ×10 magnification.

In the fluorogenic method, cells were pre-treated for 1 hour with 100 nM bafilomycin A1 (Sigma), which inhibited lysosomal acidification, and then incubated for 1 hour with 20 µM 5-dodecanoylaminofluorescein di-β-D-galactopyranoside (C_12_FDG, Sigma) a fluorogenic substrate for β-galactosidase. Cells were then detached with prewarmed 0.05% trypsin-EDTA and resuspended in PBS containing the viability stain TO-PRO-3 (Molecular probes). After 15 minutes, the measurement of cell fluorescence without (autofluorescence) and with C_12_FDG was carried out with flow cytometer (15′000 events). Data were acquired using the excitation laser at 488 nm and detection optic at 525/50 nm for both autofluorescence and C_12_FDG. A 638 nm laser and 670/30 detector was used for TO-PRO-3. Resulting data files were analyzed using FlowJo software. 15 µm polypropylene calibration beads (PHCCBEADS, Millipore) were used to standardize cytometer settings between runs (5′000 events) and dead cells were excluded from analysis.

### RNA isolation, cDNA synthesis and real time PCR

Total RNA was isolated from monolayer cultures using QIAzol Lysis Reagent (Qiagen, Hombrechtikon, Switzerland) and Direct-zol RNA MiniPrep (Zymo, LucernaChem), according to the manufacturer’s instructions. cDNA was prepared using VILO cDNA Synthesis Kit (Invitrogen) in a reaction volume of 10 µL following kit instruction, and diluted 1:10 with ultrapure water.

Real-time (RT)-PCR reactions consisted in the primers listed in Table 1 at a concentration of 250 nM, 5 µL cDNA template, and IQ SYBR Green Supermix (Bio Rad). Specific products were amplified by a quantitative PCR system (CFX96™ Real Time System, Bio Rad). RT-PCR was carried out with the following settings: denaturation 95 °C–3 minutes (1 cycle), 95 °C–15 sec, 60 °C–20 sec and 72 °C–20 sec (35 amplification cycles) in a final volume of 20 µL in 96-well plates (Bio Rad). Melting curve analysis was performed after each reaction. Relative gene expression of cyclin-dependent kinase inhibitor 2A (p16^INK4A^), 2C (p18^INK4C^) and 1 (p21^CIP1^), as well as E2F transcription factor 1 (E2F1), ankyrin repeat domain 1 (ANKRD1), cyclin D2 (CCND2), cell division cycle protein 2 homolog (CDC2) and cell division cycle associated 7 (CDCA7)^36^, was determined using the 2^−ΔΔCt^ method and the results were normalized to the expression of peptidylprolyl isomerase A (PPIA)^53^.

**Table 1:** Human genes used in quantitative RT-PCR.

hbp	Primer nucleotide sequence (5′ to 3′)	Amplicon	Efficiency
(NCBI Reference Sequence)		(bp)	(%)
**Housekeeping gene**			
PPIA^53^	F - GTCAACCCCACCGTGTTCTT	97	103.5
(NM_021130)	R - CTGCTGTCTTTGGGACCTTGT		
**Senescence associated genes**			
p16^INK4a^	F - GTGGACCTGGCTGAGGAG	132	90.1
(NM_000077)	R - CTTTCAATCGGGGATGTCTG		
p18^INK4C^	F - AGAGATCTGTAGCGTAGGTACGTG	74	104.6
(NM_078626)	R - ACATACATTCCTGGTTAATGACTCC		
p21^CIP1^	F - CGAAGTCAGTTCCTTGTGGAG	111	101.7
(NM_000389)	R - CATGGGTTCTGACGGACAT		
E2F1	F - TCCAAGAACCACATCCAGTG	75	101.2
(NM_005225)	R - CTGGGTCAACCCCTCAAG		
CDC2	F - TGGATCTGAAGAAATACTTGGATTCTA	96	102.6
(NM_001786)	R - CAATCCCCTGTAGGATTTGG		
CDCA7^32^	F - CTGCCCAGAAGCCGTCGCTC	222	126.7
(NM_031942)	R - GAACTGGCCTCGAACGCCCC		
CCND2^32^	F - CGCAACCTGCTCCGAGACGAC	228	106.6
(NM_001759)	R - AGTCGGGACCCCAGCCAAGAA		
ANKRD1^32^	F - CGGAACCTGTGGATGTGCCTACG	245	116.8
(NM_014391)	R - TCCTCCACGGCTTGCCCAGT		

(F = Forward, R = Reverse. bp = base pairs).

### DNA isolation and determination of telomere length

Telomere length was determined by RT-PCR, as previously described^54^. Briefly, genomic DNA (from 2 × 10^5^ cells) of MSC was isolated using the appropriate DNA purification kit^55^: Gentra PureGene Cell Kit (Qiagen). gDNA samples were quantified and run in triplicate in a 96-well plate in a quantitative PCR system (20 ng gDNA/well). The sequences of the both telomere and 36B4 primers are listed in Table 2. The real-time PCR program consisted of initial denaturation at 95 °C for 5 min, followed by 32 PCR cycles at 95 °C for 10 sec and 60 °C for 30 sec. Melting curve analysis was carried out for each reaction and standard curves were fitted for both telomere DNA and 36B4 DNA. For each telomere primer set, values were calculated as a ratio of telomere DNA to 36B4 DNA. In order to increase accuracy, telomere length was expressed as the average of values from the two primer sets.

**Table 2:** Human genes used in quantitative telomere PCR.

Gene	Primer nucleotide sequence (5′ to 3′)	Amplicon	Efficiency
		(bp)	(%)
**Telomere length** ^54^			
Telomere	telF - CGGTTTGTTTGGGTTTGGGTTTGGGTTTGGGTTTGGGTT	>76	72.9
	telR - GGCTTGCCTTACCCTTACCCTTACCCTTACCCTTACCCT		
36B4	F - CAGCAAGTGGGAAGGTGTAATCC	75	79.4
	R - CCCATTCTATCATCAACGGGTACAA		

(F = Forward, R = Reverse. bp = base pairs).

### Cytokine measurements

The concentrations of cytokines and chemokines were analyzed in the supernatants of MSC monoculture after 3 days incubation, by human IL-6 (Cat. No. 900-K16) and MCP-1 (Cat. No. 900-K31) ELISA development kits (Peprotech). Absorbance (405 nm, wavelength correction 650 nm) was measured using a Multimode Detector (DTX 880; Beckman Coulter) and results were quantified with standards.

### Immunoblot analyses

Whole protein content was isolated from MSC at early (low autofluorescence) and late (high autofluorescence) passages. Cells were lysed in 150 mM NaCl, 1% Triton X-100, 0.5% Na deoxicholate, 0.1% SDS, 50 mM Tris, pH 8.0, (all AppliChem) and 1X protease inhibitor cocktail (Sigma) for 30 minutes, centrifuged at 800 g for 10 min (both at 4 °C) and protein extracts (10 µg) were fractionated by Mini-Protean TGX 4–15% gradient polyacrylamide gels, and semi-dry blotted to a nitrocellulose membrane (both Bio Rad). The nitrocellulose membranes were incubated with mouse monoclonal antibodies against γ-glutamyl hydrolase (GGH; 1:250; rabbit, HPA025226, Sigma), family with sequence similarity 96, member B (FAM96B; 1:250; rabbit, HPA041736, Sigma), hepatoma derived growth factor-like-1 (HDGFL1; 1:250; rabbit, HPA045679, Sigma), and housekeeping gene β-actin (1:5000; mouse, HRP conjugate, 12262, Cell Signaling – Bio Concept). Blocked membranes were probed overnight at 4 °C with primary antibodies diluted in 1% milk (Rapilait, Migros, Switzerland) in PBS, followed by HRP-conjugated rabbit secondary antibody (1:5000, Bethyl – LuBioScience) in 1% milk in PBS, for one hour at room temperature. Membranes were developed with LumiGlo Reserve Chemiluminescent Substrate Kit (LumiGlo Reserve KPL – Bio Concept). Acquisition was performed with a digital SLR camera (Nikon D600, Nikon, Zürich, Switzerland) and the results were normalized to the relative amount of β-actin.

### MSC *in vitro* differentiation into osteogenic, chondrogenic and adipogenic lineages

MSC cultures were stimulated with the appropriate differentiation medium according to the conditions described below.

#### Chondrogenic differentiation

Collagen type I cubes (Biopad, Euroresearch, Italy) were used as scaffold to support cellular differentiation^56^. MSC (5 × 10^5^ cells) were seeded onto collagen cubes and kept for 30 minutes to allow adhesion, before addition of chondrogenic medium. MSC-collagen constructs were cultured for three weeks at 5% O_2_ in chondrogenic medium, consisting of: advanced DMEM + GlutaMAX (Gibco), 2.5% FBS, 100 units/mL penicillin, 100 mg/mL streptomycin, 2.5 µg/mL amphotericin B, 40 ng/mL dexamethasone (Sigma), 50 µg/mL ascorbic acid 2-phosphate (Sigma), 1x Insulin-Transferrin-Selenium X (Gibco), and 10 ng/ml transforming growth factor-β1 (TGF-β1, Peprotech).

Glycosaminoglycan (GAG) accumulation was determined as chondrogenic marker. GAG accumulation was quantified with alcian blue binding assay after six hours digestion of three cell-constructs per sample at 60 °C with 125 µg/ml papain (Sigma) in 5 mM L-cysteine-HCl (Fluka), 5 mM Na-citrate, 150 mM NaCl and 5 mM EDTA (all AppliChem). GAG accumulation was determined by binding to alcian blue (Fluka, Sigma) and quantified using chondroitin sulphate (Sigma) reference standards^57^.

#### Osteogenic differentiation

MSCs in monolayer at a density of 5 × 10^3^ cells/cm^2^ were differentiated in STEMPRO® Osteogenesis Differentiation Kit (Gibco) for three weeks. Calcium content was determined using the Calcium CPC LiquiColor test kit (StanBio, Schwetzingen, Germany). Cells were washed with PBS, incubated with 0.5 N HCl for 30 minutes at room temperature and then with O-Cresolphthalein complexone in alkaline solution. Calcium concentration was measured (absorbance at 405 nm) and quantified with standards.

#### Adipogenic differentiation

MSC were cultured in monolayers at a density of 2.5 × 10^4^ cells/cm^2^, alternating two different culture conditions: adipogenesis maintenance medium — DMEM/Ham’s F12+ GlutaMAX, 2.5% FBS, 1X Pen Strep, 2.5 µg/mL amphotericin B and 170 mM insulin; and adipogenesis inducing medium — adipogenesis maintenance medium supplemented with 1 µM dexamethasone, 0.5 mM 3-Isobutyl-1-methylxanthine and 0.5 mM indomethacin (all Sigma). After two weeks, lipid droplets were stained with Oil Red O (Sigma), and the dye content was quantified after isopropanol elution and spectrophotometry by measuring the absorbance at 492 nm.

### Statistical analysis

Cell volume, MSC differentiation potential markers, cytokine concentrations, telomere length and gene expression data are shown as mean values, while the data acquired at the flow cytometer is presented as geometric mean. Analysis by flow cytometry was repeated three times and results are presented as the average of three independent experiments. Kendall’s tau-b correlation - a non-parametric measure - was used to assess all correlations and *p* < *0.05* was considered significant. Correlation coefficient (τ_b_) above 0.75 was considered a high degree of correlation; correlation coefficient between 0.50 and 0.74, moderate correlation, while correlation coefficient range between 0.25 and 0.49, low degree of correlation. Absence of correlation was defined as τ_b_ lower than 0.24. In the box plots, a non-parametric test (Mann-Whitney U-test) was used to determine the significance levels. Data analysis was performed with SPSS 24.0 for Windows (SPSS Inc.).

## Data Availability

The datasets generated during and/or analyzed during the current study are available from the corresponding author on reasonable request.
